# Supplementary material for: Epstein-Barr Virus Encephalitis: A Review of Case Reports from the Last 25 Years
Source: Microorganisms. 2023 Nov 21;11(12):2825. doi: 10.3390/microorganisms11122825 (PMC10745555; doi:10.3390/microorganisms11122825)
Supplement: Supplementary file 1 [file microorganisms-11-02825-s001.zip › microorganisms-2640913-supplementary.pdf]

microorganisms MDPI

Supplementary Table S1. Viral analyses reported in each case, whether positive or negative

LCR		Blood		Ref
EBV PCR	Serology	EBV PCR	Sero-logy	
Yes	No	No	No	11, 13, 16, 21, 33, 39a, 39b, 44, 51a, 68, 75, 80, 91, 100, 103, 105, 107
Yes	Yes	Yes	No	88
Yes	Yes	No	Yes	25a, 55, 69
Yes	No	No	Yes	10, 18, 22, 24, 25b, 26, 28, 29, 30, 34, 38, 40, 43, 45, 48, 50, 51b, 53, 61, 63, 66, 67, 70, 79, 81, 84, 89, 90, 92, 96, 102
Yes	No	Yes	Yes	12, 15, 17, 19, 20, 27, 31, 32, 36, 41, 42, 54, 56, 86, 101
Yes	No	Yes	No	23, 46, 72, 77
No	No	No	Yes	14, 35, 37, 49, 59, 60, 62, 64, 65, 71, 76, 94, 97, 98, 99, 106a, 106b
No	No	Yes	Yes	47, 74, 85
No	Yes	No	No	104
No	Yes	No	Yes	52a, 52b, 52c, 95
No	Yes	Yes	Yes	73

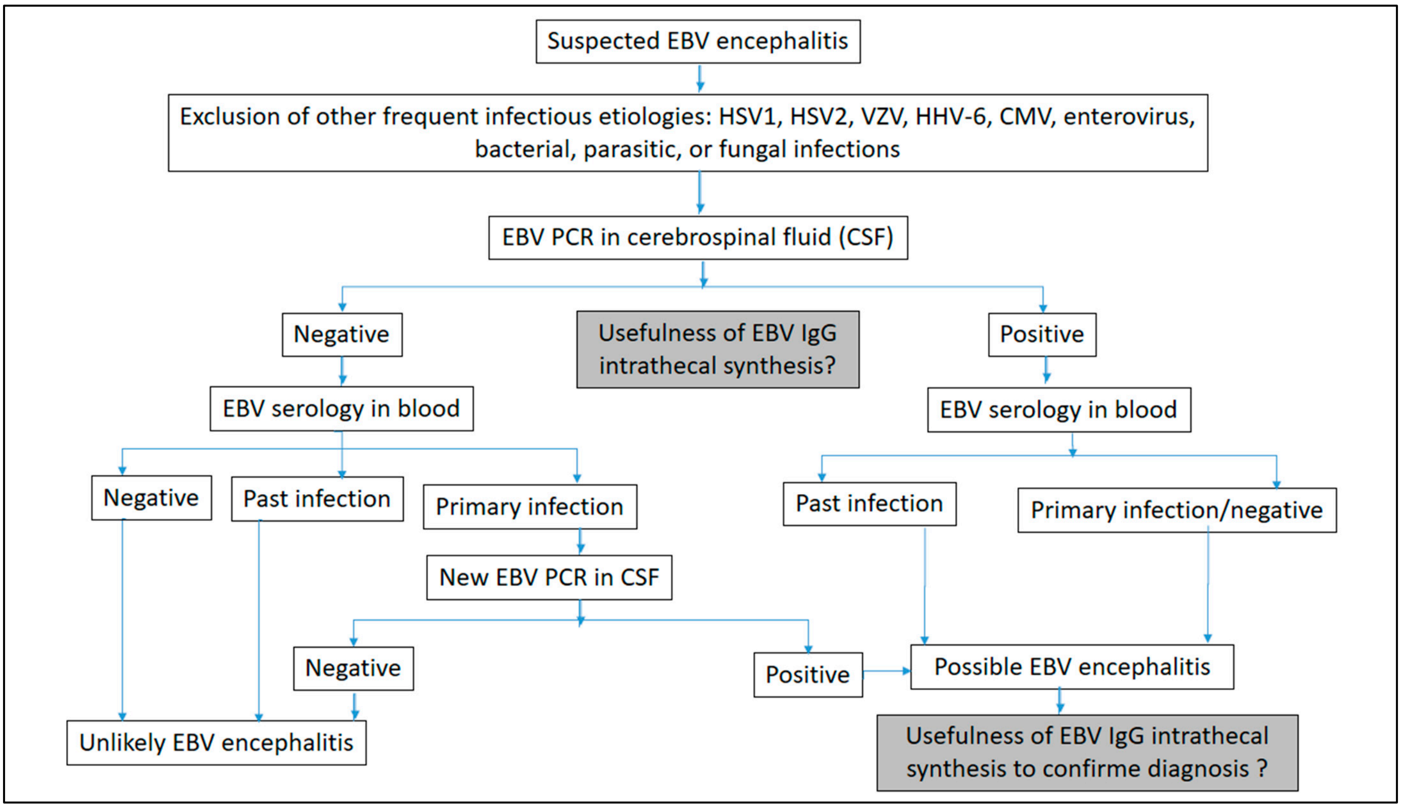

Supplementary Figure S1. Interpretation tree for virological testings in the case of suspected EBV encephalitis
